# Suppressive Mechanisms Induced by Tregs in Celiac Disease

**DOI:** 10.29252/ibj.24.3.140

**Published:** 2020-01-18

**Authors:** Nastaran Asri, Mohammad Rostami-Nejad, Mohammad Barzegar, Abdolrahim Nikzamir, Mostafa Rezaei-Tavirani, Mohammadreza Razzaghi, Mohammad Reza Zali

**Affiliations:** 1 Department of Clinical Biochemistry, School of Medicine, Shahid Beheshti University of MedicalSciences, Tehran, Iran;; 2 Gastroenterology and Liver Diseases Research Center, Research Institute for Gastroenterology and Liver Diseases, Shahid Beheshti University of Medical Sciences, Tehran, Iran;; 3 School of Medicine, Shahid Beheshti University of Medical Sciences, Tehran, Iran ;; 4 Proteomics Research Center, Faculty of Paramedical Sciences, Shahid Beheshti University of Medical Sciences, Tehran, Iran;; 5 Laser Application in Medical Sciences Research Center, Shahid Beheshti University of Medical Sciences, Tehran, Iran

**Keywords:** Celiac disease, Glutens, Immune tolerance, T-lymphocytes

## Abstract

CD is a systemic immune-mediated disorder caused by the dietary gluten in individuals who are genetically susceptible to the disease. In fact, CD is a T cell-mediated immune disease in which gluten-derived peptides activate the lamina propria CD4^+^ Teff cells, and these T-cell subsets can cause the intestinal tissue damages. Also, there are additional subsets of CD4^+^ T cells with suppressor functions. These subsets express the master transcription factor, FOXP3*,* and include Tr1 cells and CD4^+^CD25^+^ Tregs, which are the main population involved in maintaining the peripheral tolerance, preventing the autoimmune diseases and limiting the chronic inflammatory diseases such as CD. The suppressive function of Tregs is important to maintain the immune homeostasis. This paper examined the features and the basic mechanisms used by Tregs to mediate the suppression in CD.

## INTRODUCTION

The immune system tolerates all dietary antigens that enter into the body in normal conditions. In other words, feeding with these antigens induces the Tregs and tolerance associated with an immune homeostatic function of these cells^[^^1^^]^. 

CD is a chronic inflammatory disorder related to the gastrointestinal tract with the autoimmune features that are developed in genetically susceptible individuals^[^^2^^]^. The pathogenesis of CD depends on the interaction of triple factors, including genetic background, gluten ingestion, and environmental influences^[^^3^^]^. In fact, CD results from intolerance of dietary gluten peptides. The immune reaction in CD involves the adaptive as well as the innate immune responses in which the activation of lamina propria gluten-specific CD4^+^ Teff cells is a keystone of the pathogenesis of this disease^[^^4^^,^^5^^]^. Indeed, the innate and adaptive mucosal systems are firmly controlled by various regulatory circuits. For instance, the increased expression of the anti-inflammatory cytokines like IL-10 and TGF-β occurs concurrently with the release of the inflammatory factors in CD^[^^4^^-^^8^^]^. Therefore, there is a contradictory environment containing the pro- and anti-inflammatory cytokines in which regulatory mechanisms are trying to suppress the inflammation and counterbalance the gliadin-triggered, abnormal immune activation in untreated CD^[^^4^^,^^6^^-^^8^^]^. One of the most important mechanisms in suppression of inflammatory disorders such as CD is employing the Tregs^[^^9^^-^^12^^]^. This study aimed at discussing the characteristics and basic mechanisms applied by Tregs to mediate the suppression in CD.

**Tregs**

Several Tregs subsets are involved in immune tolerance. These subsets include nTregs, which originate from the thymus as CD4^+^CD25^+^ cells and are able to express the transcription factor FOXP3 and comprise about 5–10% of the peripheral CD4^+^ T cells. nTregs have already been specialized for the suppressive function before the antigen encounter. On the other hand, the adaptive or iTregs originate from CD4-positive naive T cells under immunogenic or subimmunogenic antigen stimulation in the periphery^[^^6^^,^^13^^,^^14^^]^. Meanwhile, IL-10 and TGF-β producing Tr1 cells and TGF-β producing Th3 cells are important periphery iTregs^[^^15^^]^.

The nTregs can suppress a wide range of immune cells, including those from both the innate and adaptive immune systems. They can also suppress their targets c by cell-cell contact and without producing the pro-inflammatory cytokines and accordingly do not harm their host, despite their high self-reactivity^[^^13^^,^^16^^,^^17^^]^. Unlike nTregs, most iTregs mediate their suppressive actions through cytokine-dependent pathways. Another way that nTregs choose to apply their regulatory function is through a cell-cell-mediated mechanism in which CD4^+^ T cells, CD8^+^ T cells, monocytes, antigen-presenting B cells, and DCs are targeted through the synthesis of perforin, CD18, and granzyme A in a Fas-independent manner^[^^14^^,^^17^^,^^18^^]^. Moreover, nTregs can prevent a variety of physiological and pathological immune responses to non-self-antigens, and in some cases such as organ transplantation and the treatment of the autoimmune diseases, they can establish an immunologic tolerance to non-self-antigens^[^^17^^]^. Defects in Tregs-mediated immune regulations have been associated with several autoimmune disorders. These defects may affect the number and/or function of Tregs and are different from disease to disease. These types of dysregulation can occur through multiple mechanisms even within a disease cohort^[^^19^^,^^20^^]^. 

Tr1 and FOXP3^+^ Tregs are two populations of regulatory cells increased in the intestinal mucosa of CD patients^[^^21^^-^^24^^]^. In other words, when the CD progresses and inflammatory responses are activated, Tregs are accumulated in the affected areas to counteract the Teff cells, which have an important function in CD pathogenesis. This event is in contrast to the hypothesis that the defect in Tregs' accumolation in the intestine may play a role in the pathogenesis of CD^[^^10^^-^^12^^]^.

In the initial phase of the disease, due to the presence of toxic gliadin peptides, intestinal epithelial cells are destroyed by the innate immune mechanisms. In this phase, only a slight increase in the number of Tregs is observed^[^^9^^]^. With the intervention of the adaptive immune system, the disease progresses, and massive secretion of inflammatory cytokines causes more severe epithelial cell damage. This second phase is associated with a marked increase in the number of Tregs^[^^9^^]^. However, despite the presence of Tregs, the disease continues to progress. This condition explains that the capacity of Tregs may be decreased to down-regulate native Teff cell functions, or equally, these Teff cells may fail to react to Tregs^[^^19^^,^^25^^]^. Studies have indicated that the pro-inflammatory cytokines such as IL-15 can disrupt the suppressive ability of the Tregs in active CD^[^^19^^,^^25^^]^. It has also been explained that this functional impairment of Tregs can be caused by their generation under the inflammatory conditions^[^^25^^]^. Zanzi *et al.*^[^^25^^]^ showed that CD4^+^ CD25^+^ FOXP3^+^ Tregs isolated from the intestinal samples of CD patients can potentially exert their regulatory effects *in vitro* by the suppression of IFN-γ secretion and proliferation of CD4^+^ CD25-T cells.

**Tregs markers**

Tregs express different cell surface receptors and non-receptor molecules. Among these molecules, CD25 (IL-2Rα) is the most common index for the detection of nTregs due to its high expression levels^[^^17^^]^. In various studies, the significant increased percentage of CD25^+^ T cells in lamina propria as well as increased soluble CD25 serum concentration in untreated CD patients have been observed^[^^26^^,^^27^^]^. Moreover, functional marker of nTregs is the FOXP3, which is an important regulator and a highly specific marker for development and function of Tregs^[^^17^^]^. Cosmi *et al.*^[^^16^^] ^have indicated that the genetic mutations in FOXP3-coding gene cause the fatal autoimmune disorder, named IPEX syndrome, which refers to the importance of these genes. According to Tiittanen *et al.*^[^^8^^]^ and Vorobjova *et al.*^[^^27^^]^, FOXP3 mRNA expression elevates in the small bowel mucosa of children with CD, especially in those with CD and type 1 diabetes simultaneously^[^^8^^,^^27^^]^. In fact, inflammation is considered as the main cause of this increase in FOXP3 expression^[^^8^^]^. Another study by Vorobjova *et al.*^[^^28^^]^ have demonstrated that the mucosal density of FOXP3+ Tregs enhances in CD patients compared to the healthy controls. FOXP3 and CD25 are used as well specific markers to define the nTregs and also to distinguish these cells from other T cells. The deficiency or dysfunction of nTregs can be linked to the severity of the autoimmune disease.

**Suppressive functions of Tregs**

In inflammatory diseases, Tregs are attracted to the site of inflammation and modulate the immune reaction through the direct interaction and suppression of Teff cells^[^^29^^-^^31^^]^. In general, suppressive mechanisms of Treg cells can be classified into four categories as: (1) cytolysis, (2) inhibitory cytokines, (3) metabolic disruption, and (4) modulation of APCs function^[^^6^^,^^31^^]^. All of these mechanisms need a close communication between the suppressor and the suppressed cells^[^^31^^]^. Several studies have examined some of these functions in CD^[^^32^^-^^40^^]^, but more investigation is required to elucidate other regulatory functions of Tregs in CD. 

***Suppression by cytolysis***

**Fig. 1:**
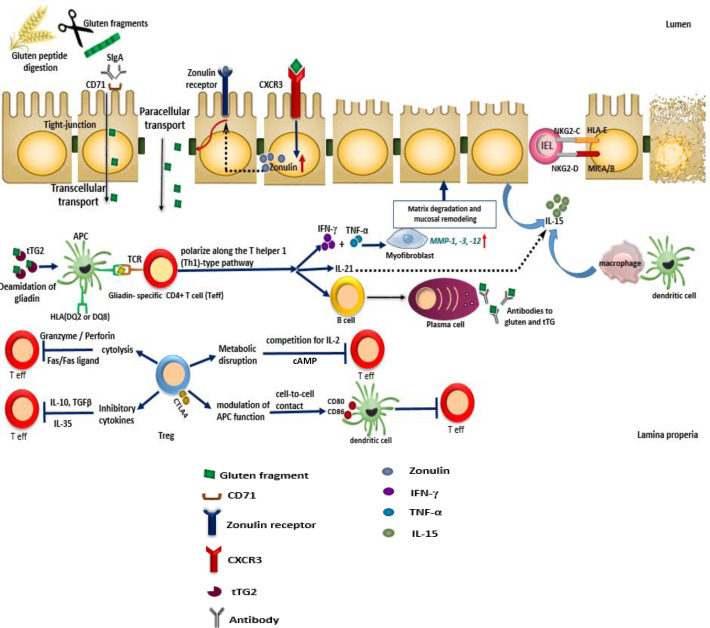
CD pathogenesis and inhibitory functions of Tregs. At first, gluten peptides pass into the lamina propria through transcellular transport across the epithelial cells by CD71 and sIgA connection or through the increased permeability of epithelial tight junctions by zonulin. In the lamina propria, tissue transglutaminase type 2 (tTG2) with deamination of gluten increases the binding affinity to HLA-DQ2 or HLA-DQ8. Hence, APCs, particularly DCs, can cause an adaptive Th1 response that will increase the production of IFN-γ, release the matrix metalloproteinases by myofibroblasts and finally result in the intestinal changes (crypt hyperplasia and villous flattening). IL-21 is another cytokine produced by CD4 Th1 T cells and often stimulates the innate immune system. Also, the density of plasma cells as well as immunoglobulin secretion increased in the lamina propria. In addition, the innate immune cells and enterocytes produced IL-15, which results in apoptosis in the intestinal mucosa with up-regulating the expression of NKG2D and NKG2C on intraepithelial lymphocytes and their ligands, MICA/B and HLA-E, on the epithelial cells. Tregs can suppress a wide range of immune cells, including Teff cells that play an important role in CD pathogenesis with four basic suppression mechanisms by: (1) cytolysis through the secretion of granzyme A/B and perforin, (2) prevention of cytokines such as IL-10, TGF-β, and IL-35, (3) metabolic disruption in methods: (a) competition for IL-2 by CD25 receptor and (b) suppression of the Teff cells by cAMP, and (4) modulation of APC function with the expression of CTLA-4(CD152) that can bind to CD80 and CD86 receptors on the DCs surfaces. Subsequently, DCs prevented the Teff cells by producing indoleamine 2,3-dioxygenase enzyme, competing with CD28 in binding to CD80/CD86 ligands, and suppressing the Teff cells activation. cAMP, cyclic adenosine monophosphate; CTLA-4, cytotoxic T lymphocyte-associated protein 4; CXCR3, C-X-C motif chemokine receptor 3; HLA DQ2/DQ8, human leukocyte antigen DQ2/DQ8; HLA-E, human leukocyte antigen E; IEL, intraepithelial lymphocyte; MICA/B, MHC class I chain-related protein A/B; MMP, matrix metalloproteinase; NKG2C, natural killer group 2C receptor; NKG2D, natural killer group 2D receptor; SIgA, secretory immunoglobulin A; RCR, T-cell receptor; tTG2, tissue transglutaminase 2

Tregs can induce the Teff cells death at the site of inflammation. This cytolysis effect can be exereted through the direct cell-to-cell contact and subsequent secretion of granzyme A/B and perforin by nTregs, and the Fas/Fas-ligand by iTregs. The granules containing granzyme and perforin are released into the extracellular space of the two reacting cells during ^[^^30^^,^^41^^]^. The perforin molecules can invade the lipid membrane of Teff cells and polymerize in the presence of calcium ions to form pores. These pores can facilitate the rapid entry of granzymes into the cell. Granzymes can also enter the cytosol via endocytosis by mannose 6-phosphate receptor. Perforin also helps to release these enzymes from endosomes. Granzymes could also bind to the cell surface and enter the Teffs by damaging the membrane^[^^42^^-^^44^^]^. It has been shown that over activation of NK cells via the perforin/granzyme pathway causes small intestinal damage in CD. However, the suppressive relevance of Tregs producing perforin/granzyme in CD remains unclear^[^^42^^]^. Granzymes can activate various death pathways. For instance, they can cause the cell death (apoptosis) in Teff cells through the caspase-dependent or -independent pathways^[^^43^^-^^46^^]^. Signaling by Fas can also result inapoptosis. These molecules can induce and maintain immune tolerance in CD by the apoptosis of gluten-reactive lymphocytes and polarization of the immune response towards protective Th2 responses^[^^30^^,^^32^^]^. De Sabatino *et al*.^[^^32^^]^ confirmed an increased Fas-mediated apoptosis in peripheral lymphocytes of untreated CD patients and believed that apoptosis occurs to preserve immune homeostasis and reduces the unwanted activity of T cells in CD^[^^32^^]^ (Fig. 1).

***Suppression by inhibitory cytokines***

Tr1 cells suppress the proliferation of pathogenic T cells by producing IL-10 and TGF-β, as anti-inflammatory cytokines, and counterbalance the gliadin-triggered abnormal immune responses in CD patients^[^^4^^,^^33^^]^. Several immune cells such as Teff cells expressing receptors for such cytokines could be influenced by the receptor-ligand interaction^[^^6^^,^^33^^]^. TGF-β can prevent the cytokine secretion by gluten-specific activated CD4^+^ T cells without the induction of their apoptosis. TGF-β also induces the production of the immunoregulatory cytokine (IL-10) in Th1 cells^[^^29^^]^.

Elevated IL-10 signaling can prevent the production of pro-inflammatory cytokines (such as IFN-γ) from Teff cells, as well as reducing the proliferation and differentiation of T cells and Teff cell function^[^^6^^,^^33^^]^. IL-10 can also interfere with antigen presentation and reduces the CD4^+^ T-cells responses to gluten in CD^[^^33^^,^^34^^]^. The other effect of IL-10 is to enhance the response of the activated T cells to TGF-β by increasing the expression of the TGF-β receptor^[^^6^^]^. IL-10 also deviates the Th1/Th2 balance to Th2 and prevents the differentiation and responses of Th1-type cells by selectively blocking IL‐12 synthesis^[^^29^^,^^33^^]^. Salvati *et al.*^[^^33^^]^ have reported that the addition of IL-10 to the intestinal biopsy culture challenged with gliadin can down-regulate the T-cell immune responses to gliadin peptides. On the other hand, increased immune responses to gliadin peptides have been observed by blocking the functions of IL-10 and TGF-β in CD patients^[^^35^^]^.

IL-35 is a heterodimeric immunosuppressor cytokine belonged to the IL-12 family and includes two chains, IL-12α (p35) and Epstein-Barr-virus-induced gene 3. Tregs are shown to express both of these chains^[^^47^^]^. IL-35 consists of two known functions contributing to Tregs suppression: the suppression of conventional T-cell proliferation and the conversion of naive conventional T cells into a strongly suppressive iTregs population, called iTr35. It has also been indicated that IL35 can convert B lymphocytes to B regulatory cells^[^^35^^,^^47^^]^ (Fig. 1). The suppressive role of IL-35 in CD immune tolerance needs more investigations. Therefore, by producing inhibitory cytokines, Tregs try to suppress the abnormal immune responses caused by gluten consumption in CD patients^[^^35^^,^^47^^]^. 

***Suppression by metabolic disruption***

*Inhibition of the proliferative response by competition for IL-2*

IL-2 is the main cytokine for T-cell proliferation. IL-2 receptor is a complex consisting of three subunits (α, β, and γ). The alpha chain of this complex (IL-2Rα) is required to increase the affinity of IL-2 to its receptor. IL-2 is required for Tregs proliferation, survival, and function. As mentioned previously, the Tregs constitutively express the high levels of IL-2 alpha chain (CD25); hence, they have a higher affinity to IL-2^[^^48^^,^^49^^]^. Since the Tregs cannot produce IL-2 and are seriously dependent on exogenous IL-2, there is a competition between these cells and the Teff cells for hiring IL-2. Therefore, the Tregs leave the Teff cells without a vital cytokine and disrupt their proliferation, causing metabolic interruption and cell death in these types of cells^[^^29^^]^. Genetic studies on autoimmune diseases have displayed that there is a region on chromosome 4q27 that contribute to IL-2 induction and has confirmed associations with CD^[^^50^^]^. The result of a meta-analysis study by Guo *et al.*^[^^50^^]^ on 12,986 CD patients and 28,733 healthy individuals showed that the allele T (rs6822844 and rs6840978) in IL2/IL21 significantly decreased the risk of CD.

*cAMP-mediated immunosuppression*

cAMP is a common intracellular second messenger that is necessary for Treg suppression. The Tregs can increase the cAMP levels in the Teff cells through two major mechanisms. The first mechanism is that the Tregs influx cAMP through the gap junctions into the Teff cells, and during the second mechanism, ATP is changed to adenosine, and this adenosine is attached to the receptors on the Teff cells surface, leading to the increased intracellular cAMP levels in these targets^[^^51^^-^^53^^]^.

Inside the Teff cells, cAMP triggers various downstream pathways that have long been associated with the inhibition of cellular proliferation and differentiation, inhibition of IL-2, and interferon-γ gene expression^[^^51^^,^^52^^]^. Peracchi *et al.*^[^^53^^]^ have reported that adenylate cyclase activity and cAMP value are significantly increased in the small intestine of CD patients compared to the control. They considered the abnormal secretion of prostaglandin E2 and hormones as the main reason for these elevated. In other study, gliadin peptides have been identified as a major contributor to the adenylate cyclase activity in the small intestinal mucosa of CD patients^[^^51^^]^. More studies are needed to highlight the role of cAMP in CD suppression.

***Suppression by modulation of APC function***

CTLA-4 (CD152), as the cell surface molecule of the Tregs, can attach to APCs stimulatory molecules (CD80 and CD86) and results in a cell-to-cell contact-dependent suppressive mechanism^[^^39^^,^^54^^,^^55^^]^. CD28 is a transmembrane protein member of the immunoglobulin gene superfamily expressed on the T-cell surface that its interaction with CD80 and/or CD86 on the APCs is essential for the complete activation of T cells. Any disturbance in this interaction can lead to T-cell non-responsiveness or anergy. CTLA-4, with higher affinity, competes with CD28 in binding to CD80/CD86 ligands and suppresses the Teff cells activation^[^^39^^,^^40^^]^. Maiuri *et al.*^[^^55^^]^ in their study used soluble CTLA-4Ig fusion in a organ culture model on gliadin challenge. Their report provided evidence that CTLA-4Ig could control CD by the suppression of pathogenic T cells. CTLA-4 interaction with CD80 and CD86 can transfer a co-stimulatory signal to the Tregs and activate them to exert the suppression mechanisms. CTLA-4 can also directly mediate the suppressive activity of the Tregs by inducing the enzyme indoleamine 2,3-dioxygenase by interacting with CD80 and CD86 in the DCs. This enzyme is a potent regulatory molecule that induces the catabolism of tryptophan into the proapoptotic metabolites, which result in the suppression of Teff cells activation^[^^54^^,^^55^^]^ (Fig. 1).

In conclusion, CD is a T-cell-mediated autoimmune condition that involves both the innate and adaptive immune responses^[^^2^^]^. Various regulatory mechanisms are formed during the course of the disease. In the most important regulatory mechanism the CD4^+^ CD25^+^ FOXP3^+^ Tregs are employed^[^^10^^-^^12^^]^. Various studies have demonstrated the marked increase of Tregs in the intestinal mucosa during the active phase of CD ^[^^12^^]^. As it mentioned, Tregs can perform their suppressive roles through the four different pathways and suppress the inflammatory disorders such as CD by affecting Teffs^[^^31^^,^^37^^,^^41^^-^^46^^,^^53^^]^. There may also be some unexplored suppressive mechanisms used by the Tregs. Numerous studies have examined some of the inhibitory functions of Tregs in CD^[^^32^^-^^40^^]^; however, other possible regulatory functions of Tregs are required to be investigated. It should be importantly noted that despite the increasing regulator and suppressor factors, CD continues to progress. It seems that some factors, such as IL-15 or Treg generation in inflammatory condition, may prevent the proper function of Tregs in CD due to unclear reasons; the Teff cells have been demonstrated to become resistant to these regulatory cells^[^^25^^,^^26^^]^. A precise identification and removal of these barriers and also an accurate examination of all suppressive mechanisms of Tregs in CD can be a main step to improve the CD treatment.
